# The PAP Gene Family in Tomato: Comprehensive Comparative Analysis, Phylogenetic Relationships and Expression Profiles

**DOI:** 10.3390/plants11040563

**Published:** 2022-02-21

**Authors:** Xin Pang, Yuan Cheng, Meiying Ruan, Qingjing Ye, Rongqing Wang, Zhuping Yao, Guozhi Zhou, Hongjian Wan

**Affiliations:** 1 Suzhou Polytechnic Institute of Agriculture, Suzhou 215008, China; pangx@szai.edu.cn; 2 State Key Laboratory for Managing Biotic and Chemical Threats to the Quality and Safety of Agro-Products, Zhejiang Academy of Agricultural Sciences, Hangzhou 310021, China; 3 Institute of Vegetables, Zhejiang Academy of Agricultural Sciences, Hangzhou 310021, China; chengyuan@zaas.ac.cn (Y.C.); ruanmy@zaas.ac.cn (M.R.); yeqj@zaas.ac.cn (Q.Y.); wangrq@zaas.ac.cn (R.W.); yaozp@zaas.ac.cn (Z.Y.); zhougz@zaas.ac.cn (G.Z.)

**Keywords:** *PAP* gene family, abiotic stress, expression profiles

## Abstract

Purple acid phosphatase (*PAP*) plays a vital role in plant phosphate acquisition and utilization, as well as cell wall synthesis and redox reactions. In this study, comprehensive comparative analyses of *PAP* genes were carried out using the integration of phylogeny, chromosomal localization, intron/exon structural characteristics, and expression profiling. It was shown that the number of introns of the PAP genes, which were distributed unevenly on 12 chromosomes, ranged from 1 to 12. These findings pointed to the existence of complex structures. Phylogenetic analyses revealed that *PAP*s from tomato, rice, and Arabidopsis could be divided into three groups (Groups I, II, and III). It was assumed that the diversity of these PAP genes occurred before the monocot–dicot split. RNA-seq analysis revealed that most of the genes were expressed in all of the tissues analyzed, with the exception of *SlPAP02*, *SlPAP11*, and *SlPAP14*, which were not detected. It was also found that expression levels of most of the SlPAP gene family of members were changed under phosphorus stress conditions, suggesting potential functional diversification. The findings of this work will help us to achieve a better insight into the function of SlPAP genes in the future, as well as enhance our understanding of their evolutionary relationships in plants.

## 1. Introduction

Phosphorus (P), a key macronutrient, is required for plant growth and development. It not only plays an important role in energy transfer and metabolic regulation, but is also is a known important structural constituent of many biomolecules, including DNA, RNA, and protein [1]. However, due to the P fixation of organic compounds and free Fe or Al oxides, the P concentration levels in soil solutions are often low. For this reason, low P availability can pose major constraints on plant growth and development [2,3]. However, plants have developed numerous strategies to adapt to low soil P concentrations, including the production of acid phosphatases (APases), the goal of which is to scavenge organic Pi, in turn enhancing P uptake [4,5,6,7].

Purple acid phosphatases (PAPs) form part of the metallophosphoesterase family, which have been identified in some bacteria, plants, and animals [8]. The researchers reported that members of PAP homologs from different plant species exhibit a conserved motif in the carboxyl end, with seven invariant amino acid residues, arranged in the five blocks (DXG/GDXXY/GNH(D/E)/VXXH/GHXH) [9]. Plant PAPs hydrolyzed phosphoric acid esters and anhydrides with an optimal pH from 4 to 7. Based on their molecular masses, they can be divided into two groups: small PAPs (i.e., monomeric proteins with molecular masses of approximately 35 kDa, structurally similar to PAPs from mammalian); and large *PAP*s (i.e., homodimeric proteins with a single polypeptide of approximately 55 kDa; these generally have two conservation domains, and are more closely homologous to enzymes from fungi and mycobacteria) [8]. 

At the present time, members of *PAP* gene family have been identified in numerous plant species. For instance, in the model plant Arabidopsis, 29 different *PAP* genes were found to be annotated and the gene transcriptions of *AtPAP11* and *AtPAP12* were found to have been induced and increased during phosphate deprivation experiments [9]. In addition, there are 26, 35, 33, and 19 members in rice [10], soybean [2], maize [11], and tea [12], respectively. In Arabidopsis, the *AtPAP10* is a Pi-starvation-induced phosphatase, is largely related with root surfaces, and plays a key role in plant resistance to phosphate limitations [13]. Furthermore, when the model plant Arabidopsis was cultivated in inorganic orthophosphate (Pi)-deficient soils, not only does *AtPAP26* aid in scavenging P from organic P sources, it also plays a vital role in P remobilization [14]. Previous research has found that *PvPAP3* was induced by Pi starvation in both the leaves and roots of common beans, and its expression levels were observed to be strictly dependent on two different conditions: phosphorus availability and the duration of the Pi-starvation conditions [15]. In addition, under typically growth conditions, *PAP*s also play important roles in both plant growth and development [16,17,18,19,20,21,22,23]. 

Previously, three Pi-starvation-responsive *PAP*s have been identified in tomato, two of which are secreted acid phosphatases (*SAP1* and *SAP2*), and one of which is an intracellular acid phosphatase (*IAP*) [24,25]. However, it was observed that *SAP1*, *SAP2*, and *IAP* had different expression patterns. In a suspension cell system, the expressions of these two genes (*SAP1* and *SAP2*) were specifically induced in response to Pi starvation. Meanwhile, the induction of *IAP* was observed on the sixth day of Pi starvation. It was observed that *SAP1* was specifically expressed in the Pi-starved roots of 30-day-old seedlings, while *IAP* was expressed in the leaves, stems, and root tissue [26]. Recently, one of the phosphate-starvation-responsive purple phosphatase, *SlPAP1*, in tomato seedlings was cloned and characterized [27]. Although previous studies characterized the potential roles of several individual *PAP* members in tomato, at present, the evolutionary relationships, expression patterns and potential functions of the members of *PAP* gene family in Pi acquisition and mobilization require further exploration. 

At present, many gene families were identified widely in different plant whole genomes [28,29,30,31,32,33]. In the current study, the *PAP* family genes in tomato were identified by integration of conserved motifs, gene structures, chromosome mapping, promoter *cis*-element and their phylogenetic relationships. These results will deepen our current understanding of the evolutionary relationships and functional divergence of the *SlPAP* genes in tomato, as well as provide some information for further comparative genomic studies throughout all of the plant family.

## 2. Results

### 2.1. Identification of the PAP Gene Family in the Tomato Genome

In the present study, a total of 18 *PAP* genes were identified and designated as *SlPAP01* to *SlPAP18*, respectively, based on the gene chromosome locations. The information on all of the *SlPAP*s (e.g., molecular weights (MW), isoelectric points (pI), numbers of exons/intros, and subcellular localization) are detailed in Table 1. It was observed that the lengths of the amino acid (aa) sequences of the candidate *SlPAP*s differed, ranging from 426 aa (*SlPAP15*) to 649 aa (*SlPAP04*), with an MW ranging from 47.9 kDa (*SlPAP15*) to 73.0 kDa (*SlPAP04*), and the pI varied from 5.80 (*SlPAP15*) to 8.22 (*SlPAP21*). The Euk-mPLoc 2.0 online tool was adopted to determine the subcellular localizations of the *SlPAP* proteins. All of the proteins were predicted to localize in the extracellular; four *PAP*s (*SlPAP04*, *SlPAP08*, *SlPAP09*, and *SlPAP10*) were also localized in the cytomembrane and ER (Table 1). Among those, the seven metal-binding residues (D, D, Y, N, H, H and H) within five consensus blocks, which belonged to the PAP metalloesterase, were conserved in 16 of 18 SlPAPs in tomato. However, the two remaining tomato PAPs (*SlPAP05* and *SlPAP18*) were observed to differ in those conserved domains of the PAPs. Furthermore, *SlPAP05* lacked the first motif, and in *SlPAP18*, one amino acid residue in the second block was observed to have been altered. However, multiple amino acid sequence alignments showed the highest homology with the known plant *PAP*s, these two genes (*SlPAP05* and *SlPAP18*) were still considered to be potential *PAP*s.

### 2.2. Phylogenetic Analysis of the PAP Genes in Tomato

**Figure 1:**
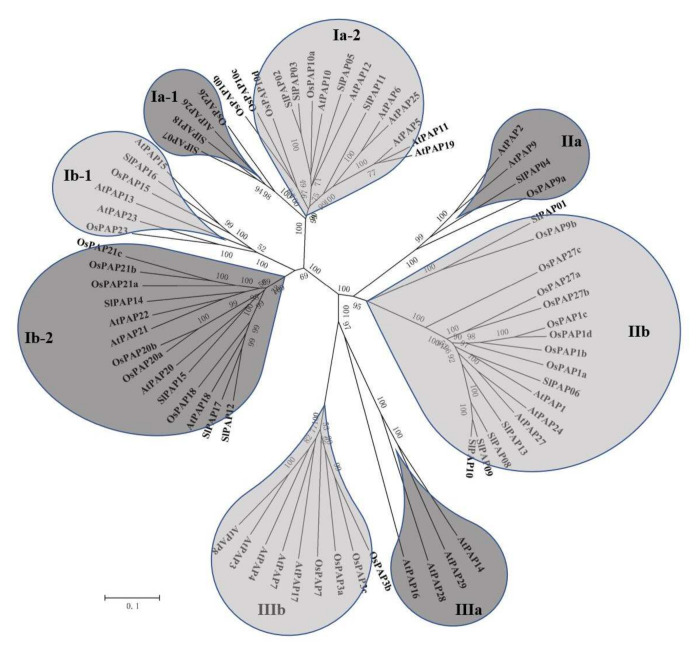
The phylogenetic tree of 73 PAP proteins from tomato, Arabidopsis and rice was constructed with MEGA5.0 software using the neighbor-joining method. Bootstrapping (1000 replicates) was used to evaluate the degree of support for each group in the phylogenetic tree.

The phylogenetic tree of the PAP proteins from tomato, Arabidopsis and rice is illustrated in Figure 1. According to the previous results [9], the 18 SlPAPs, 29 AtPAPs and 26 OsPAPs may be divided into three different groups: Groups I, II and III. Group I was subdivided into four different subgroups (Ia-1, Ia-2, Ib-1, Ib-2), and each of the remaining two groups (Groups II and III) was subdivided into two subgroups (IIa and IIb, IIIa and IIIb), respectively. The 18 SlPAPs belonged to six subgroups in the phylogenetic tree, with the exceptions of subgroups IIIa and IIIb. Subgroup IIb exhibited the most genes among the eight subgroups, including 17 PAP genes, which consisted of SlPAPs 01, 06, 08, 09, 10, and 13; AtPAPs 1, 24, and 17; and eight OsPAPs. The lowest subgroups were Ia-1, IIa, and IIIa, which included only 4 PAP genes. Subgroup Ia-1 had SlPAPs 07 and 18, AtPAP 26, and OsPAP 26. Subgroup IIa had SlPAPs, AtPAPs 2 and 9, and OsPAP 9a. Subgroup IIIa only contained AtPAPs.

This study also found seven pairs of orthologous genes between the various species: AtPAP1 and SlPAP06; OsPAP9b and SlPAP01; SlPAP05 and AtPAP12; OsPAP10a and AtPAP10; SlPAP16 and AtPAP15; AtPAP23 and OsPAP23; and SlPAP15 and AtPAP20, respectively. There were also determined to be nine pairs of paralogous genes among the species, four pairs from tomato, five pairs from rice, and nine pairs from Arabidopsis.

### 2.3. Intron/Exon Structure and Conserved Motifs of Members of SlPAP Gene Family in Tomato

**Figure 2:**
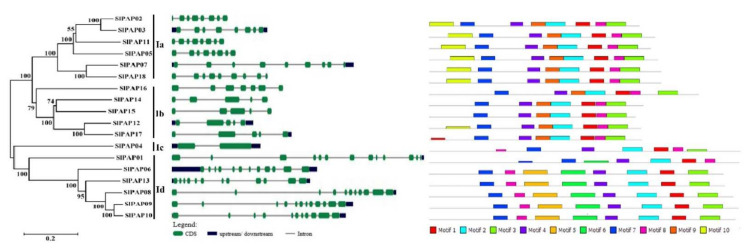
Phylogenetic analysis, intron/exon configurations and conservation motifs of PAP genes in tomato. A phylogenetic tree of PAP genes was constructed using MEGA 5.0. Introns and exons are drawn to scale with the full encoding regions of their respective genes. Boxes indicate the exon, and lines indicate the intron. 0 = intron phrase 0; 1 = intron phrase 1; 2 = intron phrase 2.

In order to investigate the phylogenetic relationships of the PAP proteins in tomato, 18 PAP proteins were identified using MEGA 5.0 software. As shown in Figure 1, the PAP proteins were divided into the four subgroups: Ia, Ib, IIa, and IIb, respectively. Intron/exon configurations of the *SlPAP* family genes were carried out by means of the online tool GSDS (http://gsds.cbi.pku.edu.cn, accessed on 4 January 2022). The numbers of introns of the remaining *SlPAP* genes ranged from 1 to 12, as detailed in Figure 2. The highest numbers were observed in *SlPAP01* and *SlPAP06*, which included 12 introns, and *SlPAP04* contained lowest number. In addition, the SlPAP genes within the same subgroups were found to contain similar numbers of introns. Subgroup Ia had 7 to 9 introns; Subgroup Ib had 4 to 6 introns; Subgroup IIa had one intron; and Subgroup IIb contained 10 to 12 introns. In total, 10 distinct motifs were identified, and 139 motifs were identified in 18 *SlPAP* genes using MEME. Every gene contained 7 or 8 motifs, and all of the subgroups had motif 1, motif 2, motif 3 (with the exception of *SlPAP01*), motif 4, and motif 7 (Figure 2). Motif 6 was only discovered in Subgroup IIb, and motif 9 and motif 10 were only present in Group I. 

### 2.4. Chromosomal Distributions and Duplications of the PAP Genes in Tomato

**Figure 3:**
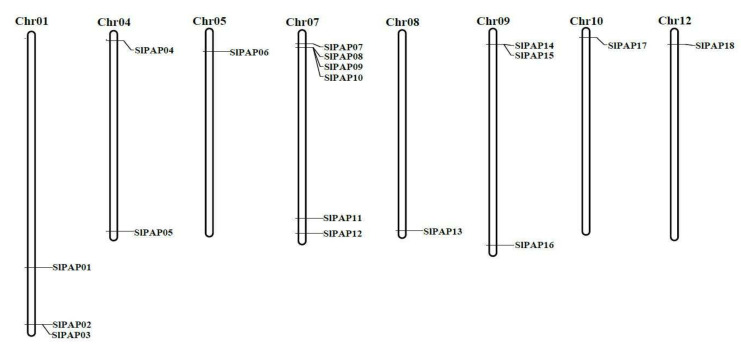
Position of SlPAP genes on the tomato chromosomes. Chromosome numbers are indicated at the top of the chromosome.

Chromosome locations showed that the SlPAP genes were distributed unevenly on eight chromosomes, including chromosome 1, 4, 5, 7, 8, 9, 10 and 12. The other chromosomes did not carry the PAP genes (Figure 3). Among these, chromosome 7 was observed to contain the greatest number of SlPAP genes, with six genes. This was followed by chromosomes 1 and 9 which contained three each. In this study, three pairs of SlPAP genes (SlPAP02 and SlPAP03; SlPAP08 and SlPAP09; SlPAP14 and SlPAP15) were shown in tandem on three chromosomes, including chromosome 1, 7 and 9. 

### 2.5. Promoter Cis-Element Analysis

**Figure 4:**
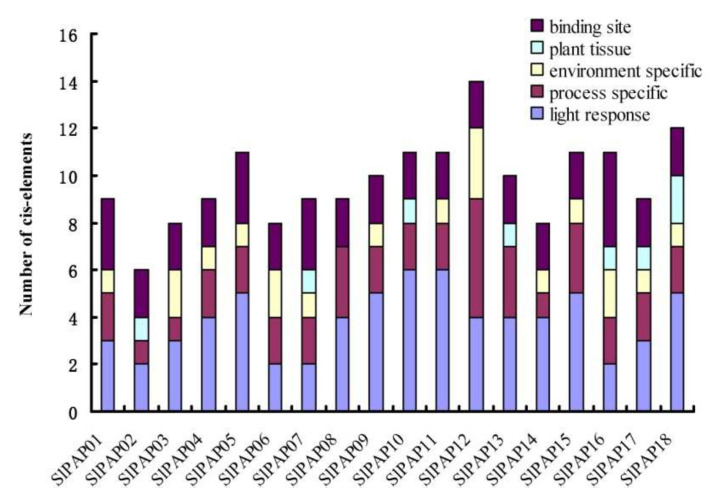
Cis-elements in the promoters of putative *SlPAP* genes that are related to stress responses. Different cis-elements with the same or similar functions are present with the same color.

In order to gain further information regarding the regulation of the SlPAP genes, this study analyzed the cis-elements of the SlPAP gene promoter sequences. The regions 1000 bp upstream from each individual SlPAP gene were obtained by means of PlantCARE. The cis-elements were divided into five major classes: light response; process-specific; environment-specific; plant tissue; and binding sites, as illustrated in Figure 4. A total of 20 light response cis-elements were identified. In addition, we also found eleven hormones-specific cis-elements, including ABA-, MeJA-, GA-, SA-, Auxin- and ethylene-responsive elements. Six types of environment-specific cis-elements were found, including ARE, CCAAT-box, LTR, and MBS, which reflected anaerobic induction, heat, low temperature, and drought, respectively. Five cis-acting elements, which were involved in tissue-specific expressions, were also found, including the AACA-motif, CAT-box, GCN4_motif, HD-Zip 1, and an O_2_-site. In addition, six binding site cis-elements were also identified.

### 2.6. Expression Analysis of Members of SlPAP Gene Family in Different Tissues and Organs Based on RNA-Seq Data

**Figure 5:**
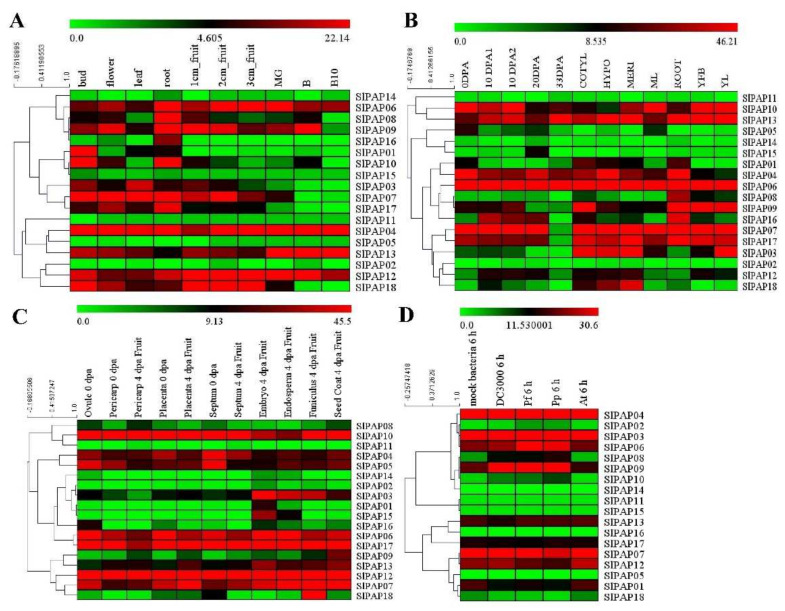
Expression profiles of *SlPAP* genes on RNA-seq in different tomato tissues and species. Heat maps are presented in green/black/red/colors that represent low/medium/high expression, respectively.

This study also analyzed the expression profiles of the candidate SlPAP genes at various tissue and developmental stages of the tomato cultivar Heinz and wild species *S. pimpinellifolium* L1589, as detailed in Figure 5A,B. The analysis results revealed that four genes (SlPAP04, SlPAP06, SlPAP12, and SlPAP13) were highly expressed, whereas five genes (SlPAP02, SlPAP05, SlPAP11, SlPAP14, and SlPAP15) were not detectable or weakly detected in all ten tissues of the Heinz tomato species. The transcripts of four genes (SlPAP04, SlPAP06, SlPAP10, and SlPAP13) were highly expressed, whereas four genes (SlPAP02, SlPAP11, SlPAP14, and SlPAP15) were not detectable or weakly detected in all the analyzed stages of the L1589. Among those genes, it was observed that SlPAP10 had the highest expression levels during the flower budding stage of the wild species *S. pimpinellifolium*. 

When the expression profiles of the SlPAP genes in the vegetative organs (leaves and roots) and reproductive organs (flower buds and flowers) between the two tomato genotypes were compared, differential expressions were observed between two genes (SlPAP12 and SlPAP18). In addition, there were differential expression profiles of four SlPAP genes in the vegetative and reproductive organs of tomato var. Heinz, while only one of those was expressed differentially in *S. pimpinellifolium* L1589. In the Heinz tomato, one SlPAP gene (SlPAP16) with the highest expression level had displayed tissue specificity in the roots, which was also observed in the *S. pimpinellifolium* L1589 variety (Figure 5A,B). The respective expression levels of two genes (SlPAP01 and SlPAP04) were found to be greatly up-regulated, whereas six genes (SlPAP03, SlPAP08, SlPAP09, SlPAP10, SlPAP13, and SlPAP16) were down-regulated during young fruit development stage (1cm_fruit, 2cm_fruit and 3cm_fruit) in the Heinz tomato cultivar (Figure 5A). It was also observed that the expression levels of five different genes (SlPAP03, SlPAP04, SlPAP12, SlPAP17, and SlPAP18) were continuously down-regulated during the fruit development stage (mature green, breaker, breaker 10) in the Heinz variety. Although SlPAP11 was observed to be continuously up-regulated, SlPAP03, SlPAP07, SlPAP12, SlPAP16, SlPAP17, and SlPAP18 were down-regulated during the fruit development stage (10DPA, 20DPA, and 33DPA) in the *S. pimpinellifolium* L1589 variety, as shown in Figure 5B. SlPAP07 and SlPAP17 were strongly expressed during the vegetative growth stage and early stage of the fruit ripening. Meanwhile, they were weakly expressed before fruit maturation in the L1589. These results indicated that those specific genes were regulated in a tissue-specific manner. 

To further deepen our current knowledge of the expression profiles of the SlPAP genes in different tissues, we analyzed the expression patterns of the SlPAP genes in the different tissues of the wild relative, S. pimpinellifolium (Figure 5C). Eight genes (SlPAP04, SlPAP05, SlPAP06, SlPAP07, SlPAP10, SlPAP12, SlPAP13, and SlPAP17) were found to be expressed constitutively throughout all of the analyzed tissues, while the expressions of three SlPAP genes (SlPAP02, SlPAP11, and SlPAP14) were at almost undetectable levels. We observed that SlPAP01 and SlPAP14 were expressed preferentially in the embryos of 4dpa fruit. In addition, the transcripts of three genes (SlPAP06, SlPAP16, and SlPAP17) in 0dpa were found to be higher than 4dpa in some of the tissue types. 

As shown in Figure 5D, eight genes (SlPAP02, SlPAP05, SlPAP10, SlPAP11, SlPAP14, SlPAP15, SlPAP16, and SlPAP18) did not have detectable expressions or had low expressions. Meanwhile, the expression levels of two genes (SlPAP03 and SlPAP04) were highly expressed for control or treatment. The expression levels of SlPAP03, SlPAP06, SlPAP08, and SlPAP09 were found to be up-regulated in response to DC3000 6h, Pseudomonas fluorescens 6h, and Pseudomonas putida 6h, and down-regulated under Agrobacterium tumefaciens 6h. However, SlPAP07 and SlPAP12 displayed completely opposite expression levels. SlPAP04 displayed up-regulated levels under Pf 6h and Pp 6h and was down-regulated under DC3000 6h and At6h. However, SlPAP12 was observed to be differently expressed. 

This study found that, by analyzing the expression profiles of the tandem duplications of SlPAP genes in the two genotypes and various types of tissue, that the two groups of SlPAP genes (SlPAP08/SlPAP09; SlPAP14/SlPAP15) had displayed a more similar expression pattern. Meanwhile, another group of SlPAP genes (SlPAP02/SlPAP03) was observed to display different expression patterns (Figure 5A,B). The expression profiles of the SlPAP genes (SlPAP02/SlPAP03; SlPAP08/SlPAP09) were determined to have different expressions, as observed in various treatment results (Figure 5D).

### 2.7. Expression Analysis of the SlPAP Genes under Phosphate Stress Treatment Condition Using qRT-PCR

**Figure 6:**
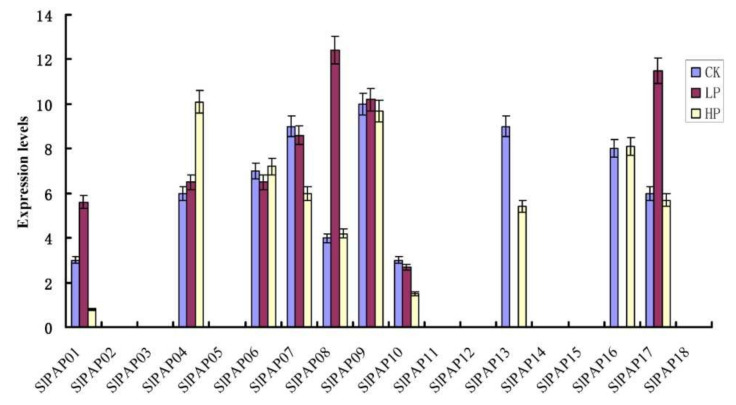
Expression profiles of *PAP* genes in tomato in response to low- and high-phosphate stress. Heat maps are presented in green/black/red/colors that represent low/medium/high expressions, respectively.

In the present study, the respective expression levels of 18 SlPAP genes were performed under both low- and high-phosphorus conditions, so as to quantify their expression levels using qRT-PCR technology. The results showed that among the 18 SlPAP genes, 8 genes (SlPAP02, SlPAP03, SlPAP05, SlPAP11, SlPAP12, SlPAP14, SlPAP15, and SlPAP18) were not detected in the control or treatments (Figure 6). The expression levels of SlPAP04 were found to be up-regulated, while four other genes (SlPAP01, SlPAP07, SlPAP10, and SlPAP13) were sown to be down-regulated, under the high-phosphorus treatments. In addition, three SlPAP genes (SlPAP01, SlPAP08, and SlPAP17) were expressed at relatively higher intensities under low-phosphorus conditions, while SlPAP06 and SlPAP16 had similar expressions under high-phosphorus conditions.

### 2.8. Interaction Networks of SlPAP Proteins

**Figure 7:**
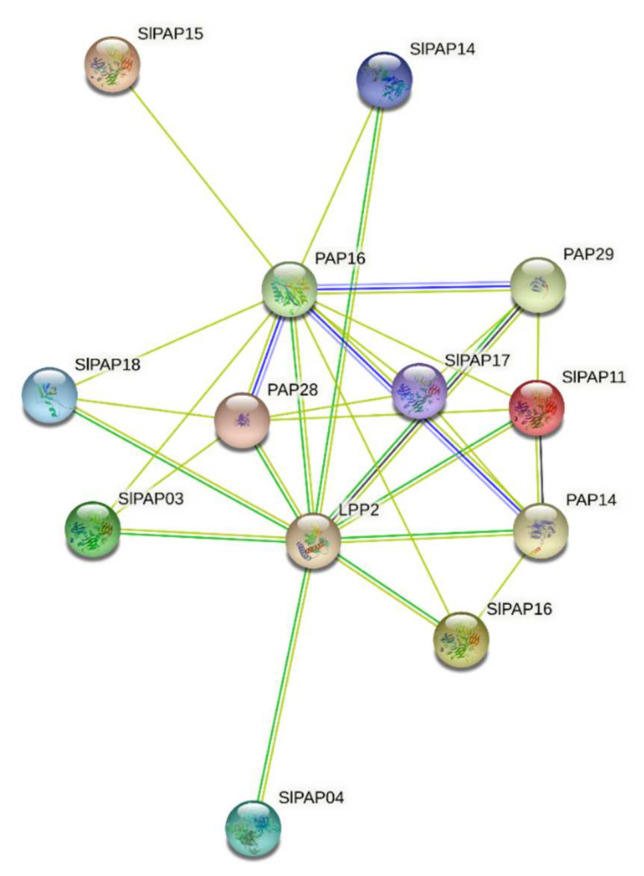
Functional interaction network of SlPAP proteins in tomato according to orthologs in *Arabidopsis*.

To understand the interactions of the SlPAP proteins, the STRING website was used to construct an interaction network. The results showed that only 8 SlPAP proteins were selected because of their reliability (Figure 7). The SlPAP17 protein, which was orthologous to PAP18 in *Arabidopsis* had more interaction partners than others. All of the proteins involving molecular function (acid phosphatase activity, GO:0003993) and cellular components (cellular anatomical entity, GO:0110165) were detected by Gene Ontology. The metal ion binding (GO:0046872) and extracellular regions (GO:0005576) were detected in most of the proteins. 

## 3. Discussion

Plant *PAP*s have diverse physiological functions, including phosphorus (P) acquisition [15,34,35]; carbon metabolism [36,37]; the generation of reactive oxygen species [38,39]; responses to abiotic stress [40,41]; involvement in flower development [16]; and cell wall biosynthesis [17,42,43]. In the past, plant *PAP*s have mainly been studied for their potential involvement in Pi nutrition. These enzymes can catalyze the hydrolysis of various phosphate esters and anhydrides within the pH range from 4 to 7 [9,44]. The majority of the PAPs are inducible by phosphate (Pi) deprivation. In recent years, the genomes of many plant species have been sequenced, including tomato. However, the numbers and structural characteristics of *SlPAP*s remain unclear at the genome level. 

**Figure 8:**
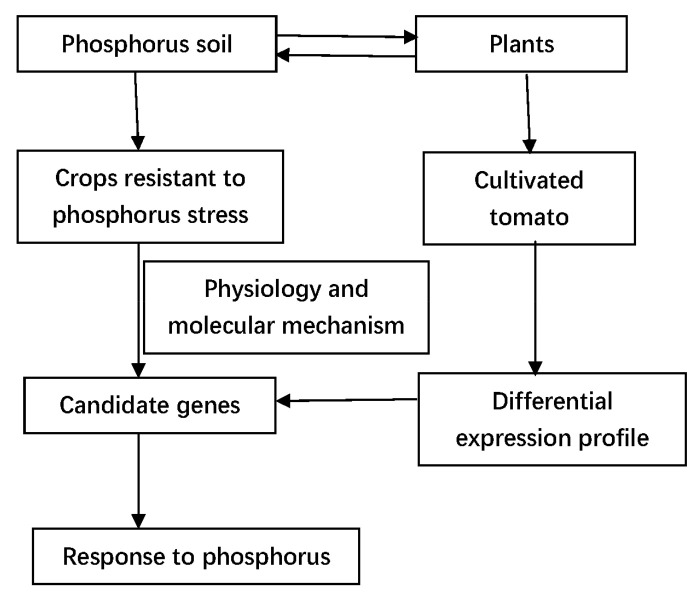
A hypothetical model of identification of SlPAP genes related to P stress using bioinformatic method.

The present study identified 18 *PAP* genes in tomato using the tomato genome database (http://solgenomics.net/, accessed on 4 January 2022) by bioinformatic method (Figure 8). The *PAP* gene family has 29 and 26 members in Arabidopsis and rice, respectively. In tomato (781 Mb), when compared with Arabidopsis (genome size 125 Mb) and rice (480 Mb), the number of the *PAP* family (18) appears small. Among the 18 *SlPAP* genes, 1 predicted *SlPAP* gene was found to have incomplete conserved domains. However, the gene was still considered to be a potential *PAP* due to its overall amino acid sequences, which exhibited the greatest homology with the known plant *PAP*s during BLASTing in NCBI. 

Classifications were made as intracellular *PAP* (IAP) and secretory *PAP*s (SAP) according to the functions of the *PAP*s. IAP is mainly used for the reuse of intracellular phosphorus and the degradation of single nucleotides, phospholipids, and so on, thereby releasing inorganic phosphorus for the normal growth of plants [45]. SAP mainly breaks down organic phosphorus substrates in soil in order to release inorganic phosphorus, which plants can directly absorb and utilize. Red kidney bean *KbPAP* is the first clear indication of the subcellular localization of plants due to its positioning in the cytoplasm [46]. In this study, Euk-mPLoc software was used to predict the subcellular localization. It was found that 14 of the 18 *SlPAP*s were only located in extracellular spaces (Table 1). Therefore, it was speculated that they may enter through the secretory pathways. This study also found that *SlPAP04*, *SlPAP09*, and *SlPAP10* were not only located in the extracellular, but also in the endoplasmic reticulum. In addition, *SlPAP08* was located in the membrane, endoplasmic reticulum, and extracellular. However, further confirmation is required to identify the functions of the aforementioned four *SlPAP* genes.

Gene duplication events are vital in the rapid expansions and evolution of gene families. In this research study, the chromosome mapping results showed that 18 *SlPAP* genes were located unevenly on eight tomato chromosomes (Figure 3). Tandem gene duplication is exhibited in many plant species, such as Arabidopsis, rice, cucumber and tomato. In this investigation, it was observed that 33.3% (6/18) of the *SlPAP* genes had evolved from tandem gene duplication. Therefore, it was considered that, in tomato, tandem gene duplication likely plays a key role in *PAP* gene family expansions. 

This study’s results also revealed that the 18 *SlPAP*s, 29 *AtPAP*s and 26 *OsPAP*s could be divided into three distinct groups and eight subgroups. The *SlPAP*s genes were only found in Groups I and II, and not Group III, which may have been due to losses in the evolutionary processes of the tomatoes. The *PAP* gene family members of tomato include six subgroups (Ia-1, Ia-2, Ib-1, Ib-2, IIa and IIb), and the same subgroups exhibited similar intron/exon structures and motif patterns. For example, four members of the Ia-2 Subgroup had 7 introns; four members of the Ib-2 Subgroup had 4 introns; and motif 6 was characteristic of Subgroup IIb, which became an important basis for the subgroup classification. Previous related studies reported that *AtPAP10*, *AtPAP12*, *AtPAP25* and *AtPAP26* are very important in the phosphorus deficiency adaptation of *Arabidopsis thaliana*. In particular, it is believed that *AtPAP25* may play a role in P-deficiency signaling as a phosphoprotein phosphatase [13,14,47,48]. It was also considered that *SlPAP02*, *SlPAP03*, *SlPAP05*, *SlPAP07*, and *SlPAP11* in the same subgroup may have similar functions. However, when combined with the quantitative expressions after P stress treatment, it was found that *SlPAP05* and *SlPAP11* were not expressed in either the control or the treatment samples. The expression levels of the other four genes decreased under low P stress conditions when compared with the control. Therefore, the function of tomato *PAP* genes could not be inferred only from the function of the Arabidopsis *PAP* genes and further research is required. 

In the current investigation, based on PlantCARE online software, the cis-elements present in the promoter sequences of the *SlPAP* genes were successfully predicted. The analyses results identified five classes of cis-elements, including light responses, as well as process-, environment-, plant-tissue-, and binding-site-related cis-elements. It was found that all of the *SlPAP* genes had more than one process-specific cis-element, including ABRE, CGTCA-motif, TGACG, GARE-motif, P-box and TATC-box, TCA-element, TGA-element and ERE. As previously stated, SA, JA, ET, and ABA play key roles in plant responses to both biotic and abiotic stress, as well as in the regulation of developmental processes [49]. In addition, fourteen genes were found to have more than one environment-specific cis-element (ARE, CCAAT-box, GC-motif, LTR, MBS, and circadian). These findings will contribute to furthering the current understanding of the various functional roles of *SlPAP* genes with regard to biotic and abiotic stress responses.

According to the RNA-Seq data, the expression patterns of the *SlPAP* genes were observed in various tissue types and developmental stages. The results showed that the expressions of 15 *SlPAP* genes were tissue- and development-specific under normal growth conditions. It was considered that all of those tissue- and development-specific expressed genes likely play vital roles in the growth and development of tomato plants, and that their functions require further investigation. In addition, the expression levels of three genes (*SlPAP04*, *SlPAP06* and *SlPAP13*) were high in all the investigated tissue types, which implied that they may be closely linked to the physiological and biochemical activities of tomato plants under normal growth conditions. Furthermore, two genes (*SlPAP12* and *SlPAP18*) exhibited differential expression levels between the cultivated tomato and wild variety *S. pimpinellifolium* in the vegetative and reproductive organs. Those findings indicated that the interspecies divergence of gene expressions may have occurred, which could potentially lead to functional specialization. 

In the present investigation, for the purpose of obtaining a greater insight into the expression patterns and putative functions of *SlPAP* genes, 18 *SlPAP* genes were evaluated under high- and low-phosphorus conditions by applying a real-time quantitative RT-PCR analysis method. In Arabidopsis, it was found that the transcript levels were reduced for *AtPAP26* during phosphatase stress, whereas the *AtPAP12* transcript levels correlated well with the relative levels of secreted *AtPAP* polypeptides [14,50]. A qRT-PCR analysis revealed that the expressions of *OsPAP10a* were specifically induced by Pi-deficiency in the shoot and root tissues. However, *OsPAP10c* was induced in the roots, yet not in the shoots [51]. *AtPAP12*, *AtPAP26*, *OsPAP10a*, *OsPAP10c*, and six *SlPAP* genes belong to the same group (Group Ia). In this study, *SlPAP02*, *SlPAP03*, *SlPAP07*, and *SlPAP18* were also observed to be significantly down-regulated by the low phosphorus treatments when compared with the control. Therefore, the results suggested that those *PAP* genes may have similar functions to those in Arabidopsis and rice. In conclusion, a comprehensive analysis of SlPAP proteins in tomato was carried out though bioinformatic method. However, the function of tomato *PAP* genes under P stress condition will be explored in the future.

## 4. Materials and Methods

### 4.1. Identification of SlPAP Genes in Tomato

In the current survey, all of the tomato genome sequence data were downloaded from the following website (http://solgenomics.net/, accessed on 4 January 2022). The local database of the tomato genome sequences was constructed by the Bioedit7.0 software. In order to retrieve all the members of the PAPs family in tomato, two methods were applied to identify the local database of the tomato genome sequences by means of the BLASTP algorithm. First, the local genome sequence database was searched according to all the members of the PAPs gene family in *Arabidopsis thaliana*, in order to obtain the homologs of PAP genes in tomato. Next, we used the Hidden Markov Model (HMM) profile of PAPs, which was from the Pfam database (http://pfam.xfam.org, accessed on 4 January 2022), as a query to retrieve this local tomato database. The putative genes that were obtained using the above technique were then further identified using the Pfam database. The conserved motif structures were then analyzed using a CDD method (http://www.ncbi.nlm.nih.gov/Structure/cdd/wrpsb.cgi, accessed on 4 January 2022). Next, the molecular weight and isoelectric point of these SlPAP proteins were predicted using the ExPASy server (www.expasy.org, accessed on 4 January 2022). 

### 4.2. Subcellular Localization, Conserved Motifs and Gene Structure Analyses of the SlPAP Proteins

In this study, the subcellular localization of the SlPAP proteins was predicted using the online Euk-mPLoc 2.0 (http://www.csbio.sjtu.edu.cn/bioinf/euk-multi-2/, accessed on 4 January 2022). Then, conserved motif analyses of the *SlPAP* genes were performed using the following parameters: The MEME (http://meme-suite.org/tools/meme, accessed on 5 January 2022) was used to number the motifs. The structural feature of the *SlPAPs* were adopted by the online tool GSDS (http://gsds.cbi.pku.edu.cn, accessed on 5 January 2022). 

### 4.3. Multiple Sequence Alignment and Phylogenetic Analysis

Each amino acid sequence of the purple acid phosphatases in the tomato genome and *PAP*s from *Arabidopsis thaliana* and rice was aligned using Clustal X (Version 1.8) and a neighbor-joining method featuring bootstrap replicates (1000) in MEGA 7.0 software. Additionally, a pairwise deletion method was applied to address any gaps or missing data in the sequences, then branch lengths were assigned via the pairwise calculations of genetic distances.

### 4.4. Chromosome Distribution of PAP Genes in Tomato

The chromosomal location of the *SlPAP* genes was determined from the SGN database. The respective positions of the *SlPAP* genes were draw on the chromosomes by implementing MapDraw V2.1 software. The duplication characteristics (tandem duplications and segmental duplications) were subsequently analyzed using the following web address: http://chibba.agtec.uga.edu/duplication/index/locus (accessed on 4 January 2022). 

### 4.5. Protein–Protein Interaction Analysis

The STRING v11.5 database (http://string-db.org, accessed on 4 January 2022) was used to analyze putative protein–protein interactions of PAP proteins. Eighteen SlPAP protein sequences were uploaded to the Multiple Proteins database, and “Organism” selected *Arabidopsos thaliana* to compare analysis. Then, using “search”, the highest-scoring proteins were used to generate the functional protein interaction network.

### 4.6. Promoter Cis-Element Analysis

The regions located 1000 bp upstream (promoter region) from each of the *SlPAP* genes were obtained from tomato SGN database. Then, the cis-elements in the promoter regions of each *SlPAP* gene were identified by means of the PlantCARE server (http://bioinformatics.psb.ugent.be/webtools/plantcare/html/, accessed on 4 January 2022) [52]. 

### 4.7. Tissue-Specific Expression Analysis

In the present study, RNA-seq data which were obtained from the Tomato Functional Genomics Database (TFGD; http://ted.bti.cornell.edu/cgi-bin/TFGD/digital/home.cgi, accessed on 4 January 2022) were used to analyze the expression profiles of the candidate *SlPAP* genes within different tissues of cultivated Heinz tomato plants (*Solanum lycopersicum*) and its wild relative, LA1589 (*S.pimpinellifolium*). The leaves were treated with various bacteria and PAMPs. Several tissues (leaves, roots, flowers and fruits) were used in the cultivated tomato plants. The fruit samples included the 1 cm, 2 cm, 3 cm, mature green, breaker, and breaker + 10 fruit. For the tomato wild species (*S. pimpinellifolium*), 10 different tissues and organs were selected for analysis purposes, namely young/mature leaves, hypocotyl, roots, cotyledons, young flower buds, anthesis flowers, 10/20-day post anthesis (DPA) fruit, and 33 DPA fruit (ripening fruit). In addition, tissue-specific transcriptome profiling of LA1589 ovaries and fruit were selected, including Ovule 0 DPA, pericarp 0 DPA, pericarp 4 DPA fruit, placenta 0 DPA, placenta 4 DPA fruit, septum 0 DPA, septum 4 DPA fruit, embryo 4 DPA fruit, endosperm 4 DPA fruit, funiculus 4 DPA fruit, and seed coat 4 DPA fruit. The various tissues were treated with different bacteria. The PAMPs included mock bacteria 6h, DC3000 6h, *Pseudomonas fluorescens* 6h, *Pseudomonas putida* 6h, and *Agrobacterium tumefaciens* 6h. Then, expression analysis of the candidate *SlPAP* family genes was performed via MultiExperiment Viewer (MeV) software [53]. 

### 4.8. Plant Materials and Growth Conditions

The tomato plants were cultivated in a growing room under 14 h light period (28 to 30 °C) and 10 h dark period (18 to 20 °C) conditions. The irrigating solution reported previously was adopted. Three biological replicates for each treatment were included in the experiments. All of the samples were immediately frozen in liquid nitrogen, after which they were stored at −80 °C for RNA isolation. 

### 4.9. RNA Isolation and Quantitative Real Time-PCR (qPCR) Analysis

The transcription levels of all the *SlPAP* genes were performed through qRT-PCR analysis. In addition, the encoding regions of the *SlPAP* genes were used to design primers based on using Primer 5.0 software. 

The total RNA isolation was performed from the treated leaves using Trizol (Tiangen, Beijing, China) and was further treated with DNase I (Transgen, Beijing, China). Then, 1 µg of each of the treated RNA samples was reverse transcribed using PrimeScriptTM RT reagent Kit for qRCR (Transgen, Beijing, China). The primer information of the *SlPAP* genes is listed in Supplementary Table S1. Furthermore, qRT-PCR reactions were performed in a total volume of 20 µL including 10 µL of SuperMix, 1 µL of each of the primers, 1 µL of the template, 7 µL of sterile distilled H_2_O, and 1 µL of the passive reference dye. The thermal conditions reported previously were adopted. Finally, the 2^△△Ct^ method was used to calculate the relative gene expression values. The tomato housekeeping gene GAPDH was used as an endogenous control for the purpose of normalizing the samples [54]. qRT-PCR analyses were carried out using three biological and technical replicates.

## Figures and Tables

**Table 1:** General information about the 18 *SlPAP*s genes.

Gene	Gene Accession No.	Chromosome Location	Length of Protein	Predicted Size (kDa)	pI	No.of Exons/Intros	Subcellular Localization
*SlPAP01*	Solyc01g068380.2.1	ch01:77582424..77592344	640	71.3	5.85	13/12	extracellular
*SlPAP02*	Solyc01g110050.1.1	ch01:96853466..96855663	433	49.7	7.04	8/7	extracellular
*SlPAP03*	Solyc01g110060.2.1	ch01:96857667..96861427	466	53.2	6.34	8/7	extracellular
*SlPAP04*	Solyc04g005450.2.1	ch04:310785..314269	649	73.0	6.67	2/1	ER, extracellular
*SlPAP05*	Solyc04g080920.1.1	ch04:64973575..64976085	472	54.2	6.41	8/7	extracellular
*SlPAP06*	Solyc05g012260.2.1	ch05:5546798..5552510	607	68.2	6.41	13/12	extracellular
*SlPAP07*	Solyc07g007670.2.1	ch07:2316679..2323829	478	54.8	6.60	10/9	extracellular
*SlPAP08*	Solyc07g008550.2.1	ch07:3488895..3497719	627	71.0	6.53	11/10	Cytomembrane, ER, extracellular
*SlPAP09*	Solyc07g008560.2.1	ch07:3500707..3507834	637	72.3	6.68	12/11	ER, extracellular
*SlPAP10*	Solyc07g008570.2.1	ch07:3522999..3529845	631	71.7	6.70	11/10	ER, extracellular
*SlPAP11*	Solyc07g053070.1.1	ch07:61511946..61514007	457	52.8	6.82	8/7	extracellular
*SlPAP12*	Solyc07g064500.2.1	ch07:66617664..66620861	437	49.5	5.89	5/4	extracellular
*SlPAP13*	Solyc08g083250.2.1	ch08:65763315..65768754	609	68.6	6.08	12/11	extracellular
*SlPAP14*	Solyc09g009600.1.1	ch09:3016831..3020600	442	50.4	6.20	5/4	extracellular
*SlPAP15*	Solyc09g009610.1.1	ch09:3023290..3027216	426	47.9	5.80	5/4	extracellular
*SlPAP16*	Solyc09g091910.1.1	ch09:71122299..71126678	556	63.2	5.84	7/6	extracellular
*SlPAP17*	Solyc10g006300.2.1	ch10:950380..955091	439	49.6	6.85	5/4	extracellular
*SlPAP18*	Solyc12g009800.1.1	ch12:3005117..3008886	478	55.3	8.22	9/8	extracellular

## Data Availability

All of the related sequence data in this study were downloaded from public database and detailed in Material and Methods.

## Supplementary Materials

The following supporting information can be downloaded at: https://www.mdpi.com/article/10.3390/plants11040563/s1, Table S1: The primer sequences used in this study.

## References

[1] Duff S.M.G., Sarath G., Plaxton W.C. (1994). The role of acid phosphatases in plant phosphorus metabolism. Physiol. Plant..

[2] Li C.C., Gui S.H., Yang T., Walk T., Wang X.R., Liao H. (2012). Identification of soybean purple acid phosphatase genes and their expression responses to phosphorus availability and symbiosis. Ann. Bot..

[3] Marschner H. (1995). Mineral Nutrition of Higher Plants.

[4] Abel S., Ticconi C.A., Delatorre C.A. (2002). Phosphate sensing in higher plants. Physiol. Plant..

[5] Goldstein A.H., Danon A., Baerlein D.A., McDaniel R.G. (1988). Phosphate starvation inducible metabolism in *Lycopersicon esculentum*: II. Characterization of the phosphate starvation inducible-excreted acid phosphatase. Plant Physiol..

[6] Liu C.M., Muchhal U.S., Uthappa M., Kononowica A.K., Raghothama K.G. (1998). Tomato phosphate transporter genes are differentially regulated in plant tissues by phosphorus. Plant Physiol..

[7] Raghothama K.G. (1999). Phosphate acquisition. Annual review of plant physiology and plant molecular biology. Plant Biol..

[8] Olczak M., Morawiecka B., Watore W. (2003). Plant purplr acid phosphatases-genes, structures and biological function. Acta BiBiochim. Pol..

[9] Li D.P., Zhu H.F., Liu K.F., Liu X., Leggewie G., Udvardi M., Wang D.W. (2002). Purple acid phosphatases of *Arabidopsis thaliana*. Comparative analysis and differential regulation by phosphate deprivation. J. Biol. Chem..

[10] Zhang Q., Wang C., Tian J., Li K., Shou H. (2011). Identification of rice purple acid phosphatases related to phosphate starvation signaling. Plant Biol..

[11] González-Muñoz E., Avendaño-Vázquez A., Montes R.A., Folter S.D., Andrés-Hernández L., Abreu-Goodger C., Sawers R.J.H. (2015). The maize (*Zea mays* ssp. *mays* var. B73) genome encodes 33 members of the purple acid phosphatase family. Front. Plant Sci..

[12] Yin C.Y., Wang F., Fan H.Q., Fang Y.M., Li M.F. (2019). Identification of tea plant purple acid phosphatase genes and their expression responses to excess iron. Int. J. Mol. Sci..

[13] Wang L.S., Li Z., Qian W.Q., Guo W.L., Gao X., Huang L.L., Wang H., Zhu H.F., Wu J.W., Wang D.W. (2011). The Arabidopsis purple acid phosphatase AtPAP10 is predominantly associated with the root surface and plays an important role in plant tolerance to phosphate limitation. Plant Physiol..

[14] Robinson W.D., Carson I., Ying S., Ellis K., Plaxton W. (2012). Eliminating the purple acid phosphatase AtPAP26 in *Arabidopsis thaliana* delays leaf senescence and impairs phosphorus remobilization. New Phytol..

[15] Liang C.Y., Tian J., Lam H., Lim B.L., Yan X.L., Liao H. (2010). Biochemical and molecular characterization of PvPAP3, a novel purple acid phosphatase isolated from common bean enhancing extracellular ATP utilization. Plant Physiol..

[16] Zhu H.F., Qian W.Q., Lu X.Z., Li D.P., Liu X., Liu K.F., Wang D.W. (2005). Expression patterns of purple acid phosphatase genes in Arabidopsis organs and functional analysis of *AtPAP23* predominantly transcribed in flowers. Plant Mol. Biol..

[17] Kaida R., Hayashi T., Kaneko T.S. (2008). Purple acid phosphatase in the walls of tobacco cells. Phytochemistry.

[18] Herman V.D. (2012). Biochemical and Molecular Characterization of AtPAP25, A Novel Cell Wall-Localized Purple Acid Phosphatase Isozyme Upregulated by Phosphate-Starved *Arabidopsis thaliana*. Master’s Thesis.

[19] Law Y.S., Zhang R.S., Guan X.Q., Cheng S.F., Sun F., Duncan O., Murcha M.W., Whelan J., Lim B.L. (2015). Phosphorylation and dephosphorylation of the presequence of precursor MULTIPLE ORGANELLAR RNA EDITING FACTOR3 during import into mitochondria from Arabidopsis. Plant Physiol..

[20] Zhang R.S., Guan X.Q., Law Y.S., Sun F., Chen S., Wong K.B., Lim B.L. (2016). AtPAP2 modulates the import of the small subunit of Rubisco into chloroplasts. Plant Signal. Behav..

[21] Ping T.L., Chao Z., Shan W.S., Jia Y., Sheng Z.X., Hua S.Y. (2017). FUSCA3 interacting with LEAFY COTYLEDON2 controls lateral root formation through regulation *YUCCA4* gene expression in *Arabidopsis thaliana*. New Phytol..

[22] Sun Y.Z., Law Y.S., Cheng S.F., Lim B.L. (2017). RNA editing of cytochrome c maturation transcripts is responsive to the energy status of leaf cells in *Arabidopsis thaliana*. Mitochondrion.

[23] Zhu X.L., Lee S.Y., Yang W.T., Lee S.W., Baek D.W., Li M.S., Kim D.H. (2019). The Burholderia pyrrocinia purple acid phosphatase *PAP9* mediates phosphate acquisition in plants. J. Plant Biol..

[24] Bozzo G.G., Raghothama K.G., Paxton W.C. (2002). Purification and characterization of two secreted purple acid phosphatase isozymes from phosphate-starve tomato (*Lycopericon esculentum*) cell cultures. Eur. J. Biochem..

[25] Bozzo G.G., Raghothama K.G., Plaxton W.C. (2004). Structural and kinetic properties of a novel purplr acid phosphatase from phosophate-starved tomato (*Lycopericon esculentum*) cell cultures. Biochem. J..

[26] Bozzo G.G., Dunn E.L., Plaxton W.C. (2006). Differential synthesis of phosphate-starvation inducible purple acid phosphatase isozymes in tomato (*Lycopericon esculentum*) suspension cells and seedlings. Plant Cell Environ..

[27] Suen P.K., Zhang S.Y., Sun S.S. (2015). Molecular characterization of a tomato purple acid phosphatase during seed germination and seedling growth under phosphate stress. Plant Cell Rep..

[28] Kumar M., Kherawat B.S., Dey P., Saha D., Singh A., Bhatia S.K., Ghodake G.S., Kadam A.A., Kim H., Manorama (2021). Genome-wide identification and characterization of PIN-FORMED (PIN) Gene family reveals role in developmental and various stress conditions in *Triticum aestivum* L.. Int. J. Mol. Sci..

[29] Tong T., Fang Y.X., Zhang Z.L., Zheng J.J., Zhang X., Li J., Niu C.Y., Xue D.W., Zhang X.Q. (2021). Genome-wide identification and expression pattern analysis of the *KCS* gene family in barley. Plant Growth Regul..

[30] Pooja M.B., Debasish B.K., Kuntala S.B., Sarvajeet S.G., Niraj A. (2021). Genome wide identification and characterization of abiotic stresss responsive IncRNAs in *Capsicum annuum*. Plant Physiol. Biochem..

[31] Kesawat M.S., Kherawat B.S., Singh A., Dey P., Kabi M., Debnath D., Saha D., Khandual A., Rout S., Manorama (2021). Genome-wide identification and characterization of Brassinazole-resistant (*BZR*) Gene Family and Its expression in the Various Developmental stage and Stress Conditions in Wheat (*Triticum aestivum* L.). Int. J. Mol. Sci..

[32] Jiang M., Chen H., Liu J., Du Q., Lu S., Liu C. (2021). Genome-wide identification and functional characterization of natural antisense transcripts in *Salvia miltiorrhiza*. Sci. Rep..

[33] Wang M., Chen B.W., Zhou W., Xie L.N., Wang L.S., Zhang Y.L., Zhang Q.Z. (2021). Genome-wide identification and expression analysis of the *AT-hook Motif Nuclear Localized* gene family in soybean. BMC Genom..

[34] Tomscha J.L., Trull M.C., Deikman J., Lynch J.P., Guiltinan M.J. (2004). Phosphatase under-producer mutants have altered phosphorus relations. Plant Physiol..

[35] Lung S.C., Leung A., Kuang R., Wang Y., Leung P., Lim B.L. (2008). Phytase activity in tobacco (*Nicotiana tabacum*) root exudates is exhibited by a purple acid phosphatase. Phytochemistry.

[36] Sun F., Carrie C., Law S., Murcha M.W., Zhang R.S., Law Y.S., Suen P.K., Whelan J., Lim B.L. (2012). AtPAP2 is a tail-anchored protein in the outer membrane of chloroplasts and mitochondria. Plant Signal. Behav..

[37] Sun Q.Q., Li J.Y., Cheng W.Z., Guo H.H., Liu X.M., Gao H.B. (2018). *AtPAP2*, a unique member of the PAP family, functions in the plasma membrane. Genes.

[38] Del Pozo J.C., Allona I., Rubio V., Leyva A., de la Peña A., Aragoncillo C., Paz-Ares J. (1999). A type 5 acid phosphatase gene from *Arabidopsis thaliana* is induced by phosphate starvation and by some other types of phosphate mobilizing/oxidative stress conditions. Plant J..

[39] Liao H., Wong F.L., Phang T.H., Cheung M.Y., Li W.Y., Shao G., Yan X.L., Lam H.M. (2003). GmPAP3, a novel purple acid phosphatase-like gene in soybean induced by NaCl stress but not phosphorus deficiency. Gene.

[40] Zhang W.Y., Gruszewski H.A., Chevone B.I., Nessler C.L. (2008). An Arabidopsis purple acid phosphatase with phytase activity increases foliar ascorbate. Plant Physiol..

[41] Bhadouria J., Singh A.P., Mehra P., Verma L., Srivastawa R., Parida S.K., Giri J. (2017). Identification of purple acid phosphatases in chickpea and potential roles of *CaPAP7* in seed phytate accumulation. Sci. Rep..

[42] Kaida R., Satoh Y., Bulone V., Yamada Y., Kaku T., Hayashi T., Kaneko T.S. (2009). Activation of β-glucan synthases by wall-bound purple acid phosphatase in tobacco cells. Plant Physiol..

[43] Kaida R., Serada S., Norioka N., Norioka S., Neumetzler L., Pauly M., Sampedro J., Zarra I., Hayashi T., Kaneko T.S. (2010). Potential role for purple acid phosphatase in the dephosphorylation of wall proteins in tobacco cells. Plant Physiol..

[44] Oddie G.W., Schenk G., Angel N.Z., Walsh N., Guddat L.W., de Jersey J., Cassady A.I., Hamilton S.E., Hume D.A. (2000). Structure, function, and regulation of tartrate-resistant acid phosphatase. Bone.

[45] Veneklaas E.J., Lambers H., Bragg J., Finnegan P.M., Lovelock C.E., Plaxton W.C., Price C.A., Scheible W., Shane M.W., White P.J. (2012). Opportunities for improving phosphorus-use efficiency in crop plants. New Phytol..

[46] Cashikar A.G., Kumaresan R., Rao N.M. (1997). Biochemical characterization and subcellular localization of the red kidney bean purple acid phosphatase. Plant Physiol..

[47] Veljanovski V., Vanderbeld B., Knowles V.L., Snedden W.A., Plaxton W.C. (2006). Biochemical and molecular characterization of AtPAP26, a vacuolar purple acid phosphatase up-regulated in phosphate-deprived Arabidopsis suspension cells and seedlings. Plant Physiol..

[48] Del Vecchio H.A., Ying S., Park J., Knowles V.L., Kanno S., Tanoi K., She Y., Plaxton W.C. (2014). The cell wall-targeted purple acid phosphatase AtPAP25 is critical for acclimation of *Arabidopsis thaliana* to nutritional phosphorus deprivation. Plant J..

[49] Vision T.J., Daniel G.B., Steven D.T. (2000). The Origins of Genomic Duplications in Arabidopsis. Science.

[50] Tran H.T., Qian W.Q., Hurley B.A., She Y.M., Wang D.W., Plaxton W.C. (2010). Biochemical and molecular characterization of AtPAP12 and AtPAP26: The predominant purple acid phosphatase isozymes secreted by phosphate-starved *Arabidopsis thaliana*. Plant Cell Environ..

[51] Tian J.L. (2013). Functional Analysis of Genes in Rice Purple Acid Phosphatase Ia Subgroup. Master’s Thesis.

[52] Chou K.C., Shen H.B. (2010). A new method for predicting the subcellular localization of eukaryotic proteins with both single and multiple sites: Euk-mPLoc 2.0. PLoS ONE.

[53] Howe E., Holton K., Nair S., Schlauch D., Sinha R., Quackenbush J., Ochs M.F. (2010). Mev: Multiexperiment viewer. Biomedical Informatics for Cancer Research.

[54] Løvdal T., Lillo C. (2009). Reference gene selection for quantitative real-time PCR normalization in tomato subjected to nitrogen, cold, and light stress. Anal. Biochem..

