# Supplementary material for: The PAP Gene Family in Tomato: Comprehensive Comparative Analysis, Phylogenetic Relationships and Expression Profiles
Source: Plants (Basel). 2022 Feb 21;11(4):563. doi: 10.3390/plants11040563 (PMC8879926; doi:10.3390/plants11040563)
Supplement: Supplementary file 1 [file plants-11-00563-s001.zip › plants-1565526-supplementary.pdf]

Table S1 The primer sequences used in this study

Name	Forward primer	Reverse primer	Amplicon Length (bp)/Tm	annealing temperature(℃)
SlPAP01	GTGCACCTGTTCATGCTGTT	TTGTTGCATGAACTCGCACA	123/79.3	50
SlPAP02	GCACGTAGCACGAACTTTCT	TACGAACAACACTGCTTGCC	143/78.6	52
SlPAP03	TCAGAGCATCAGACAGCACA	GAACAATCAGCCACGGAGTC	151/78.5	52
SlPAP04	AGGAATTTACTCGCCACCCA	TCTCCGATTCCGTCCATCTG	144/79.6	52
SlPAP05	GCGATCATGAAGGAAACGGT	CCCTCGGCAAAGCTCTTAAC	92/76.8	52
SlPAP06	CGTGGACAACAGACTCGTTC	GCACCACACAAGCTACTACG	65/77.0	54
SlPAP07	TGGCTAGTGGAGCAAAGAGT	TCCCAGCAGACCAAATCCAT	150/78.5	52
SlPAP08	CCATGGGAAGGGAGCATTTG	GACTTCCTCCTCCACCAACA	189/78.3	54
SlPAP09	ATTCGGGTGGAGAATGTGGT	TGCAAAGCATTGCTCGATGA	176/78.2	52
SlPAP10	GCTTTGCGTCAGCCAATAGA	ATGAACGTGCCCGAAGAATG	185/79.1	52
SlPAP11	TGACATGGTGTTTGCAGGAC	AGTAGCTTGGCTGTGGTTCT	191/78.9	52
SlPAP12	GAAGGTTTAGCGCGCAAGTA	GCTCCAAGAGGCATGAGTTG	114/79.5	52
SlPAP13	AGACAGACAGAAGCAGCCTT	TTCCCATGGGCTCCTCAAAT	107/79.7	52
SlPAP14	ACGCCACCTTCAACATTTCC	AGTGGTTGTTGTGTGTCAGC	155/79.4	52
SlPAP15	AGGCAACCATGAGGTTGAGA	ATCAGTGTACGAGCCCAACA	166/78.5	52
SlPAP16	TAATGCGGGAGGCATACACT	TACCAAGGTGGATGCCAAGT	150/79.0	52
SlPAP17	TGATTTGGGCCAGACTGGAT	TAGAACTAGCAAGCGGCTGA	158/78.6	52
SlPAP18	GTTGAGCCAAGCACAGCATA	GACGCCCTCCTGATGGAATA	176/78.9	52
